# Supplementary material for: Autoinhibition in the Signal Transducer CIN85 Modulates B Cell Activation
Source: J Am Chem Soc. 2023 Dec 19;146(1):399–409. doi: 10.1021/jacs.3c09586 (PMC10786037; doi:10.1021/jacs.3c09586)
Supplement: Supplementary file 1 — ja3c09586_si_001.pdf [file ja3c09586_si_001.pdf]

Supplementary Information

to the article

"Autoinhibition in the Signal Transducer CIN85 Modulates B Cell Activation"

by

Daniel Sieme[1], Michael Engelke[2], Nasrollah Rezaei-Ghaleh[3,4], Stefan Becker[1], Jürgen Wienands[2] and Christian Griesinger[*,1]

[1] Department for NMR-based Structural Biology, Max Planck Institute for Multidisciplinary Sciences, Am Fassberg 11, 37077 Göttingen, Germany

[2]Institute for Cellular and Molecular Immunology, Georg-August University Göttingen, Humboldtallee 34, 37073 Göttingen, Germany.

[3]Institute of Physical Biology, Heinrich Heine University Düsseldorf, Universitätsstraße 1, 40225 Düsseldorf, Germany.

[4]Institute of Biological Information Processing, IBI-7: Structural Biochemistry, Forschungszentrum Jülich, Wilhelm-Johnen-Straße, 52428 Jülich, Germany.

Supplementary Methods

Peptide Synthesis

The peptides used during the course of this work were synthesized by means of solid-phase peptide synthesis with an acetylated N-terminus and amidated C-terminus. A LiChrosorb RP-18 column (MerckMillipore) was used for the purification and peptides were dissolved in demineralized H_2O with 0.1% trifluoroacetic acid (TFA) as a running buffer prior to injection. Elution was performed with a linear gradient of 0-100% (v/v) acetonitrile with 0.1% TFA. The purity was checked to be larger than 95% by liquid chromatography-electrospray mass spectrometry. After lyophilization the peptides were stored at -20°C until needed.

Sample preparation for in vitro experiments

$CIN85_{1-333}$, was expressed and purified as described previously[1]. Briefly, it was expressed in *Escherichia coli* strain BL21(DE3) (New England Biolabs) as a fusion protein with a N-terminal His_7-tag. The proteins were first purified by metal affinity chromatography (Protino Ni-NTA, Macherey Nagel). The His_7-tag was removed by proteolytic cleavage with TEV protease and the proteins were finally purified by size exclusion chromatography on a HiLoad 16/60 SD 75 gel filtration column (GE Healthcare). The $CIN85_{163-333}$ construct was generated based on the $CIN85_{1-333}$ construct using the primers 5'-GCG TCT AGA GGA TCC GGC ATT TCC CAG GAT GAG CAG-3' and 5'-GTC CTC GAG AAG CTT TAT TCC TTT TCA AAG TCC GGT GGA AGT AAC-3'. The R227A, R229A, S230A and S230D mutations were then introduced into the corresponding construct by PCR-based site-directed mutagenesis (Quikchange II, Agilent) and expressed and purified with the same protocol as for $CIN85_{1-333}$. The individual SH3 domains were expressed in *Escherichia coli* BL21(DE3) (New England Biolabs). For CIN85 SH3A and SH3B, the coding sequences were cloned into a pGEX-4T vector, which encodes a fusion protein with an N-terminal glutathione-S-transferase (GST) affinity tag and a Thrombin cleavage site. After cell lysis the proteins were purified by affinity chromatography using Pierce®Glutathione Agarose. The final purification step was accomplished by means of size exclusion chromatography using a HiLoad®16/600 Superdex®75 pg (GE Healthcare) chromatography column. The purified protein was then transferred to the final buffer containing 20mM HEPES pH 7.2, 100mM NaCl, 1.0mM TCEP and 0.5mM Pefabloc using dialysis. Aliquots were stored at -80°C. For CIN85-SH3C, the coding sequence was cloned into a modified pET16b vector encoding a N-terminal His_7 affinity tag and a tobacco mosaic virus (TEV) protease cleavage site. Purification was carried out using the protocol described for $CIN85_{1-333}$ above. The sample was dialyzed into the same buffer as for SH3A and SH3B and aliquots were stored at -80°C. For samples that required isotopic labeling, bacteria were cultured in M9 minimal medium with ^{15}N-NH_4Cl and/or $^{13}C_6$-D-glucose as the nitrogen and carbon source, respectively. For samples requiring additional perdeuteration, the expression was carried out in D_2O-based M9 minimal medium.

Cells, expression vectors, Ca^{2+} monitoring, and immunopurification

DG75 B cells were cultured in RPMI 1640 supplemented with 10% fetal calf serum, 2 mM L-glutamine, 1 mM sodium pyruvate, 50 µM 2-mercaptoethanol and antibiotics. B cell receptor (BCR) stimulation of the cells was performed with 0.5µg/mL - 2µg/mL of F(ab')2-anti-human IgM (Jackson ImmunoResearch) at 37°C. For the generation of Cbl-interacting protein of 85 kDa (CIN85) and CD2AP-double-deficient cells we used CRISPR/Cas9-mediated genome editing using the sg-RNAs GAT GAA TTA ACT ATT CGA GTT GG (targeting exon 2 of CD2AP) and CCC TGC ACG AAG TGC CCA GTG GA (targeting exon 3 of SH3KBP1). For reconstitution, the cDNAs encoding human CIN85 and variants, respectively, were cloned into the pMSCV vector (Clontech) containing the sequence for the EGFP variant citrine, resulting in N-terminal citrine-tagged proteins, and allowing for retroviral transduction. For flow cytometric monitoring of the BCR-induced Ca^{2+} mobilization, cells were stained with Indo-1 and analysed as described previously[2-3]. For purification of citrine-CIN85 or variants for mass spectrometry analysis, 2×10^8 cells were lysed in lysis buffer (50 mM TRIS/HCl pH 7.5, 150 mM NaCl, 1.0 mM EDTA, 0.5% Deoxycholate, 0,1% SDS; 1% (v/v) NP40; 1.0 mM Na_3VO_4, 1 tablet cOmplete®-EDTA free (Roche)) for 1 h. Samples were cleared by 10 min centrifugation at 20,000 × g. Supernatants were incubated with GFP selector beads (Nano-Tag). Beads were washed 6 times with lysis buffer and treated with lithium dodecyl sulfate (LDS) sample buffer at 70°C for 10 min.

Imaging Flow Cytometry

CIN85/CD2AP-deficient DG75 cells expressing citrine-CIN85 and the citrine-CIN85-S230A variant, respectively, were analyzed by imaging flow cytometry (Luminex ImageStream X MKII). For evaluating the differences in droplet formation, 3000 cells were monitored in $n > 10$ experiments. For analysis via the Ideas software (Luminex), a mask was generated using the feature "dots" (M=2,Ch2, bright 39 82 3.1) and based on this mask the drop count feature was utilized. The proportion of cells having $n > 0$ of the defined spots was determined. For the analysis of BCR-induced recruitment of CIN85 proteins, 1500 cells were measured without or 3min after BCR engagement. Based on the "feature finder" tool of the Ideas software, recruited cells were identified using the "spot count (Ch02)" and "H variance mean (Ch02)" features. For further analysis, the proportion of cells with recruited CIN85 was determined based on the "spot count" high and "H variance mean" for $n > 15$ experiments.

Mass Spectrometric Analyses

Mass spectrometric analyses were performed by the Core Facility Proteomics at the University Medical Center Göttingen. Samples were reconstituted in 1×NuPAGE LDS Sample Buffer (Invitrogen) and run into 4-12% NuPAGE Novex Bis-Tris Minigels (Invitrogen) for 1 cm distance. Gels were stained with Coomassie Blue for visualization purposes, and each sample cut out as a whole and diced. After washing, gel slices were reduced with dithiothreitol (DTT), alkylated with 2-iodoacetamide and digested with the endopeptidase Trypsin (sequencing grade, Promega) overnight. The resulting peptide mixtures were then extracted, dried in a SpeedVac, reconstituted in 2% acetonitrile/0.1% formic acid/ (v:v) and prepared for nanoLC-MS/MS as described previously[4].

For mass spectrometric analysis samples were enriched on a self-packed reversed phase C18 precolumn (0.15 mm ID x 20 mm, Reprosil-Pur120 C18-AQ 5 µm, Dr. Maisch, Ammerbuch-Entringen, Germany) and separated on an analytical reversed phase-C18 column (0.075 mm ID × 200 mm, Reprosil-Pur 120 C18-AQ, 3 µm, Dr. Maisch) using a 30 min linear gradient of 5-35% acetonitrile/0.1% formic acid (v:v) at 300 nL min^{-1}. The eluent was analyzed on a Q Exactive hybrid quadrupole/orbitrap mass spectrometer (ThermoFisher Scientific, Dreieich, Germany) equipped with a FlexIon nanoSpray source and operated under Excalibur 2.5 software using data-independent acquisition (DIA). Each experimental cycle contained one full mass spectrometry (MS) scan across the 350-1600 *m/z* at a resolution setting of 70000 full width at half maximum (FWHM), and AGC target of 1×10^6 and a maximum fill time of 60msec, and 11 variable window size MS/MS segments recorded at a normalized collision energy setting of 25%, a resolution setting of 17500 FWHM, an AGC target of 1×10^6 and a maximum fill time of 60msec. Three technical replicates per sample were acquired.
DIA-MS data were processed using Spectronaut version 16.0 (Biognosys, Schlieren, Switzerland). Proteins were identified using the Pulsar search engine at default settings against the UniProtKB human reference proteome v2021.01 along with a set of common lab contaminants. Protein quantitation was achieved by integrating up to 6 fragments per precursor, and up to 10 precursors per protein at 1.0% false discovery rate (FDR), respectively. Protein abundance values were quartile normalized and used for further statistical analysis.

NMR Spectroscopy

NMR Titration experiments

For the NMR titration experiments, the isolated SH3 domains were diluted from the concentrated aliquots to the desired concentration into buffer containing 20mmol HEPES, 100mM NaCl, 1mM TCEP, 0.5mM Pefabloc®SC, 5% D_2O and 0.5mM DSS as the chemical shift standard. The samples were finally transferred to 3mm NMR tubes (Hilgenberg GmbH, Malsfeld). The lyophilized peptides were dissolved in 20mmol HEPES, 100mM NaCl and the pH was again adjusted to 7.2. Standard ^1H-^{15}N HSQC experiments (Bruker pulse sequence hsqcetf3gpsi2) were collected at every titration point and the peptide was added successively to the sample, ensuring sufficient mixing before acquiring each experiment. 2D spectra were in general apodized by a cosine-squared function in both dimensions and processed using the NMRPipe software[5] (10.9 Rev 2021.258.11.26 64-bit). Dissociation constants were obtained by a global nonlinear least-squares fit of the obtained CSP of residues exhibiting fast-exchange to a two-state binding model with the following equation[6]:

$$\Delta\delta_{obs} = \Delta\delta_{max} \; \frac{(([P]+[L]+K_D)-([P+L]+K_D)^2-4[P][L])^{\frac{1}{2}}}{2[P]} \qquad (1)$$

These calculations were performed in Python 3.7.1 using the lmfit package v1.0.2[7]. Errors in the resulting dissociation constants were determined by performing a bootstrapping error calculation

with 1000 resampled datasets with replacement. After obtaining the dissociation constant, the Gibbs free energy of this interaction was calculated using the following relation:

$$\Delta G^0 = RTln(K_D) \quad (2)$$

with R as the ideal gas constant (8.314 $Jmol^{-1}K^{-1}$) and T as the absolute temperature in K. This quantity is useful when comparing two similar interactions that differ in their K_D, i.e. variants of the same peptide that have a single-point mutation or a post-translational modification (PTM) and exhibit differential binding. Their difference in free energy can then be calculated as follows:

$$\Delta\Delta G = G_1^0 - G_2^0 \quad (3)$$

$$\Delta\Delta G = RTln\left(\frac{K_D^1}{K_D^2}\right) \quad (4)$$

The value of $\Delta\Delta G$ here has the meaning of the stability contribution or net energetic contribution of the residue that was mutated.

Backbone resonance assignment of CIN85 linker regions

3D experiments for the resonance assignment of CIN85 linker regions were acquired using magnetic field strengths from 700-1200 MHz (see Supplementary Tables S1&S2). Spectra were sparsely sampled to 5-10% of the total points using poisson-gap schedules generated via http://gwagner.med.harvard. edu/intranet/hmsIST/gensched_new.html. Spectral reconstruction was carried out using SMILE[8] or hmsIST[9]. Inspection of the processed spectra was done using POKY[10]. The iterative semi-automated assignment was accomplished using FLYA[11] with the custom experiment definitions for the ^{13}C-detected experiments used listed below. The buffer condition for the samples used for the backbone resonance assignment was as follows: 20 mM MES pH 6.0, 100 mM NaCl, 1.0 mM TCEP, 0.5 mM DSS, 5 vol% D_2O, 0.5 mM EDTA and 0.01% NaN_3.

Determination of rotational correlation times and translational diffusion coefficients

Residue-specific cross-correlated relaxation rates were determined at 800 MHz via the 2D TRACT experiment[1, 12-13], using variable delays from 1-190 ms. The number of scans was varied depending on the protein construct and concentration to obtain an acceptable SNR (NS between 8-64). Rates were fitted using a single exponential model. Calculation of apparent rotational correlation time (τ_c) values from the cross-correlated η_{xy} rates was done using a published python script from Robson *et al.*[14].

The N-TRO-STE diffusion experiments[15] were acquired at 800 MHz. Translational diffusion coefficients (D_{tr}) were obtained through fitting the pulsed-field gradient-based intensity attenuation data to Stejskal-Tanner (ST) equation using a non-linear least squares approach[15]:

$$I = I_0 \exp[-D_{tr}Q] \quad (5)$$

with $Q = \gamma_H^2 g^2 \delta^2 (\Delta - \frac{\delta}{3})$. γ_H is the gyromagnetic ratio of ^{1}H, g is the peak amplitude of the gradient pulse, and Δ and δ are the durations of the long diffusion delay and the short bipolar gradient pulses, respectively. Δ and δ were chosen as 70 ms and 3.7-5.95 ms, respectively. The buffer condition for the samples used for TRACT and N-TRO-STE experiments was as follows: 20 mM HEPES pH 7.2, 100 mM NaCl, 1.0 mM TCEP, 0.5 mM DSS, 5 vol% D_2O, 0.5 mM EDTA and 0.01% NaN_3.

Arginine sidechain MQ-CEST experiments

The arginine sidechain MQ-CEST experiments introduced by Karunanithy *et al.*[16] were acquired for a reference sample of 100 mM free $^{13}C_6$,$^{15}N_4$]-L-arginine (25 mM HEPES, pH 5.1, 10% D_2O) and for $CIN85_{163\text{-}333}$ and its R229A mutant. The buffer condition for the protein samples used for MQ-CEST experiments was as follows: 20 mM MES pH 6.0, 100 mM NaCl, 1.0 mM TCEP, 0.5 mM DSS, 5 vol% D_2O, 0.5 mM EDTA and 0.01% NaN_3. All experiments were acquired at a magnetic field strength of 800 MHz and at 278 K, using the ^{1}H-detected version of the pulse sequence yielding the $^1H^{\varepsilon}$-$^{15}N^{\varepsilon}$ correlation[16]. The ^{15}N CEST elements (T_{CEST}) were 250 ms long and ^{15}N B_1 fields of 11.8 Hz, 14.6 Hz, 17.33 Hz, 20.1 Hz and 22.9 Hz were used for the free arginine sample. For the protein samples, ^{15}N B_1 fields of 11.8 Hz, 17.3 Hz and 22.9 Hz were used. The ^{15}N B_1 fields were calibrated as described elsewhere[17]. The ^{15}N carrier offsets for both the free arginine and the protein samples were chosen to span the chemical shift range between 63.3-80.1 ppm using 69 evenly spaced steps of 20 Hz. One reference spectrum per ^{15}N B_1 field value was acquired, setting T_{CEST} equal to zero. For the following analysis, peak intensities were quantified using NMRPipe (10.9 Rev 2021.258.11.26 64-bit)[5] and intensity ratios (I/I_0) were calculated based on the respective peak intensity in the reference spectrum. The least-square fitting procedure was done according to Karunanithy *et al.*[16], providing rates of rotation (k_{ex}) around the N□-C□ bond and the chemical shift difference ($\Delta\omega$) between the two N^{η} nuclei of arginine sidechain guanidinium groups. The arginine sidechain $^1H^{\varepsilon}$-$^{15}N^{\varepsilon}$ cross peaks were assigned in $CIN85_{163\text{-}333}$ based on the correlations from the backbone amide resonances to the sidechain resonances in a 3D HSQC-NOESY spectrum. The spectrum was acquired using the noesyhsqcf3gpwg3d pulse sequence from the standard Bruker pulse sequence library (τ_{mix} = 150 ms). The size of time-domain was chosen as 2048/64/128 (^{1}H/^{15}N/^{1}H) points for the direct and the two indirect dimensions, respectively. The experiment was acquired at 900 MHz and 283 K. NUS was used to reduce the amount of time-domain points that needed to be sampled, amounting to 41% of the full matrix. Spectral processing and NUS reconstruction was accomplished as described above. Analysis of the spectrum was done using POKY[10].

Resonance Assignment of the IDR containing CIN85-PRM1

To study the intramolecular CIN85-PRM1/SH3C interaction, observing the binding induced chemical shift changes and the expected line broadening of CIN85-PRM1 resonances, their

assignments are necessary. We initially used ^{1}H-detected experiments to assign the complete flexible linker regions in uniformly [^{13}C/^{15}N]-labeled CIN85$_{1\text{–}333}$. The different multi-domain constructs of the CIN85 protein used throughout this work are schematically shown in Supplementary Figure S5. Due to extensive line-broadening in the vicinity of CIN85-PRM1 in particular and the high proline content in the linker regions in general, we were not able to assign them completely (25% and 87% sequence coverage for the first and second linker region, respectively). The line-broadening observed specifically in the vicinity of CIN85-PRM1 may be due to exchange with water and between bound and unbound states of the motif. In addition, the bound state is likely experiencing a slowing-down of rotational tumbling, leading to increased transverse relaxation rates. Indeed, the correlations to residues within the folded domains were also not accessible by these experiments due to their large rotational correlation time τ_c.

We addressed this problem in two ways: firstly, by reducing the system size and looking at the linker region of interest separately, and secondly by switching to ^{13}C-detected experiments that can directly detect proline residues and avoid intermediate-exchange line-broadening effects that are especially strong for ^{1}H resonances. Additionally, ^{13}C-detection allowed increasing the temperature from 283 K to 298 K without suffering from increased solvent-exchange.

The construct containing the second linker region, CIN85$_{163\text{–}333}$, was stable and allowed for near-complete assignment of backbone and sidechain resonances using 3D ^{13}C-detected experiments. Despite lacking the SH3B domain at its N-terminus, the majority of resonances were unperturbed compared to the longer CIN85$_{1\text{–}333}$ construct (Supplementary Figure S14 and S15). By this approach, 97% of the linker backbone resonances, including most aliphatic sidechains, were assigned. However, this excluded the correlations to the residues L226, R227 and R229 in the 3D spectra, which belong to the core of CIN85-PRM1 and were likely broadened beyond detection (see Figure 3C in the manuscript).

The R229A and R227A/R229A mutations likely perturb the interaction in multi-domain CIN85 constructs, based on their effects observed in SH3C-peptide titrations (Figure 2 in the manuscript). Therefore, we applied the ^{13}C-detected approach discussed above to assign the CIN85$_{163\text{–}333}$ constructs carrying these mutations. The full backbone assignment was obtained for the R227A/R229A mutant, while the ^{1}H^N resonances of L226 and R227 were not accessible in the R229A mutant. The resonance assignment for all three constructs were deposited as BMRB entries 52079, 52080 and 52081.

The experimental parameters of the triple-resonance experiments utilized in this study can be found in Supplementary Table S1 and Supplementary Table S2.

Determination of Effective Concentrations

We utilized the 'c_{eff} Calculator' webserver[18] to estimate this parameter for the SH3C:PRM interaction. We used the backbone and sidechain chemical shifts of the region between residues 163-269 determined for the CIN85$_{163\text{–}333}$ construct to generate dihedral angles using TALOS-N[19]. These dihedral angles were then used to generate an ensemble of 30 structures of a peptide ranging from residue 219-232, utilizing the error-distribution in the predicted ϕ/ψ angles from TALOS-N to model the heterogeneity of the resulting peptide structure. We then used HADDOCK[20-21] to

dock the peptide to the SH3C domain (pdb: 2k9g), using the residues of the SH3C domain showing significant CSP in the NMR titration experiment as 'active' residues (residues 281, 282, 284, 285, 303, 304, 306, 317, 321, 322). In the peptide, we used both proline and arginine residues as 'active' residues (P223, R227, P228, R229). For the resulting structures in the four best clusters determined by HADDOCK based on the Z-score, distances between residues 232 at the C-terminal end of the docked peptide and the end of the linker at the SH3C domain (K269) were determined. This was based on the approach detailed in Kjaergaard *et al.*[18] for the determination of effective concentrations in an autoinhibited complex. In total, 16 complex structures were analyzed in this way and this resulted in the spread of potential effective concentrations ranging from 1.3-6.3 mM, depending on the corresponding contact distances shown in Supplementary Figure S12. From the derived effective concentrations and the dissociation constant of the interaction between the untethered CIN85-PRM1 and the SH3C domain obtained from the NMR titration experiments (see Table 1 in the manuscript), we calculated the equilibrium constant of this intramolecular interaction K_{intra} using Equation 6. Following this we further determined the populations of the bound and the unbound state by applying Equations 7 and 8, respectively. The corresponding bar plot illustrating the populations of the two states is shown in Supplementary Figure S11. We found the population of the closed state ranging from 0.97-0.86 going from the highest determined local concentration to the lowest.

$$K_{intra} = \frac{c_{eff}}{K_D} \quad (6)$$

$$f_{bound} = \frac{K_{intra}}{1+K_{intra}} \quad (7)$$

$$f_{unbound} = \frac{1}{1+K_{intra}} \quad (8)$$

Supplementary Figures

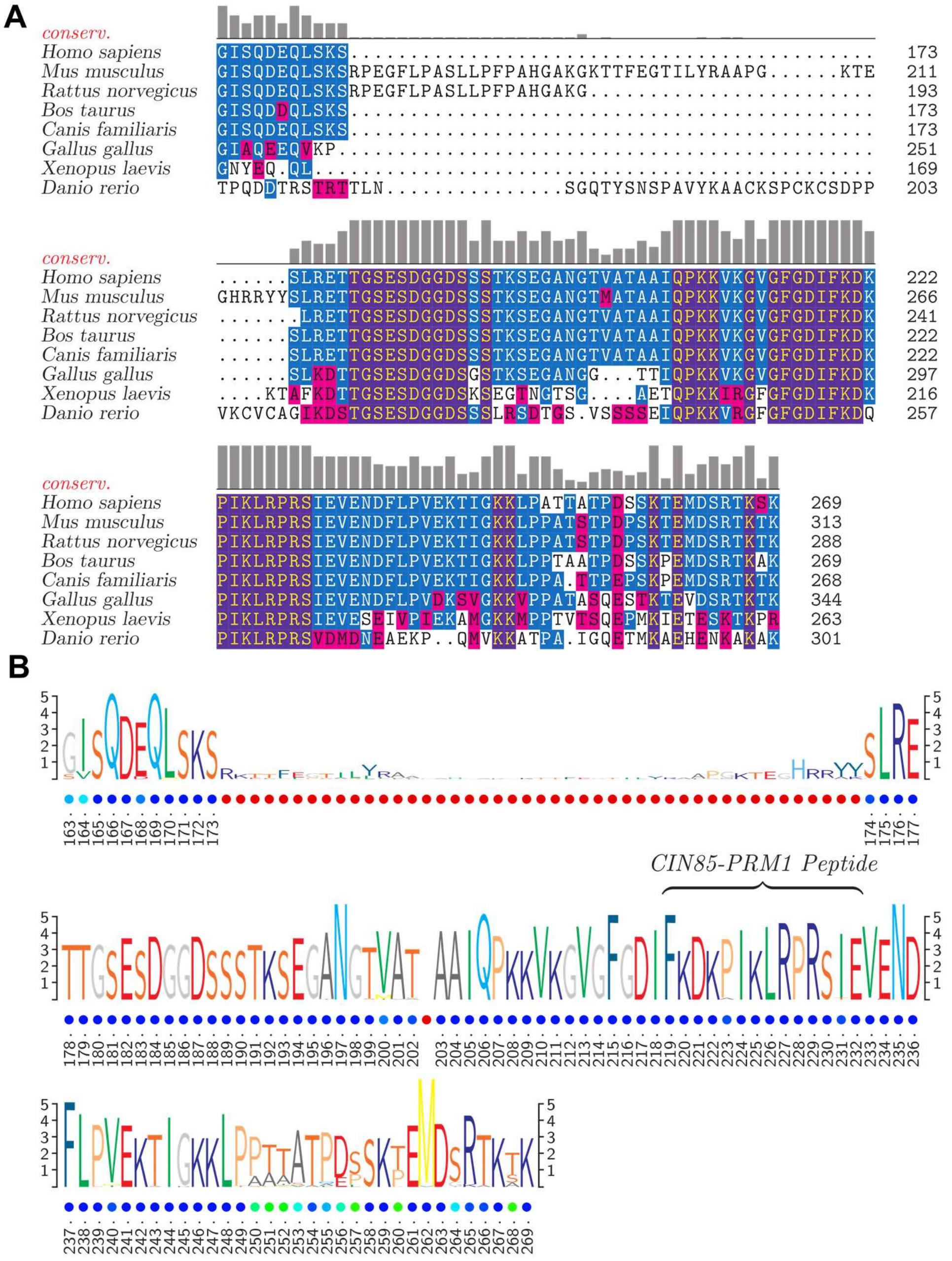

Supplementary Figure S1: A: Multiple sequence alignment (MSA) of 7 representative CIN85 homologs showing the degree of sequence conservation in the linker region between SH3B and SH3C (residues 163-269). The MSA was generated with ClustalOmega[22] based on a pBLAST[23] search of the CIN85 Uniprot entry Q96B97-1 (referenced to residues 200-239). The sequence conservation was plotted as bars on the top of each amino acid position. **B:** Sequence logo of residues 163-269 from the human protein sequence based on the first 100 sequences from non-redundant species of the pBLAST search, normalized to the natural frequency of each amino acid.

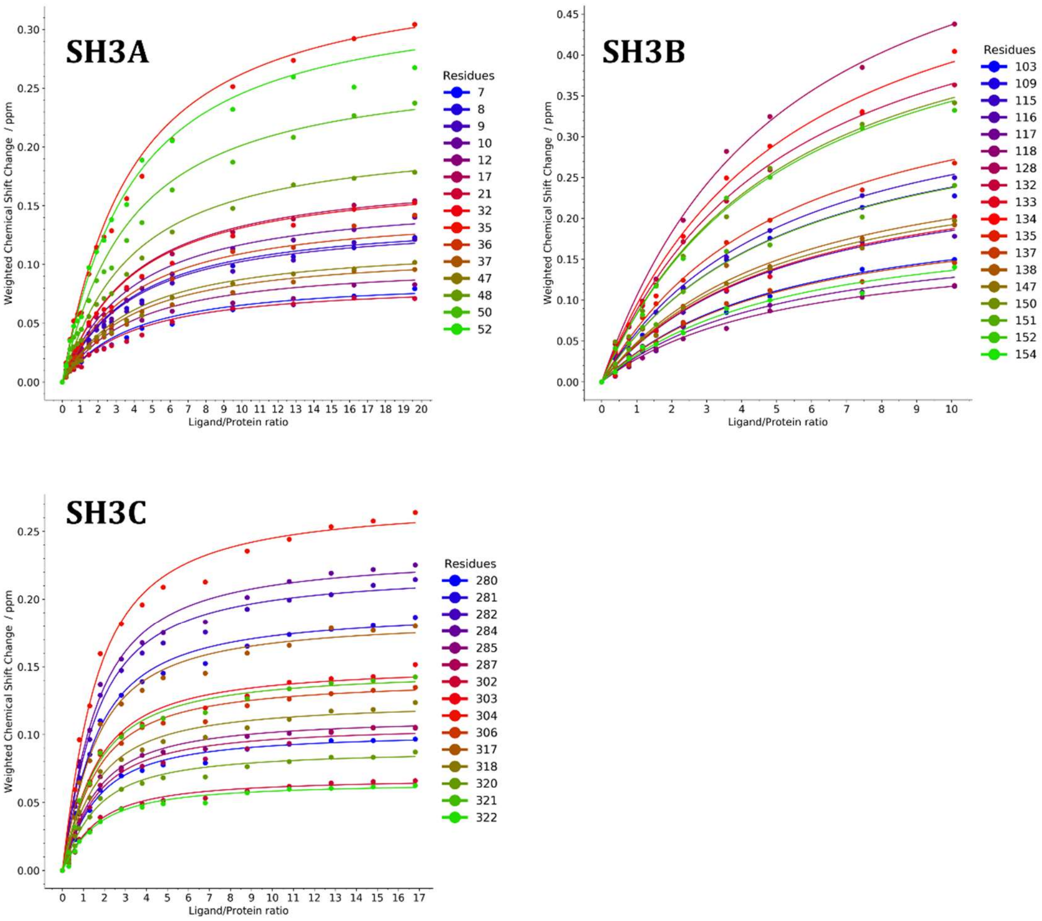

Supplementary Figure S2: Global fits of chemical shift perturbation (CSP) curves obtained after titration of ^{15}N-labeled CIN85 SH3A-C domains with increasing amounts of CIN85-PRM1.

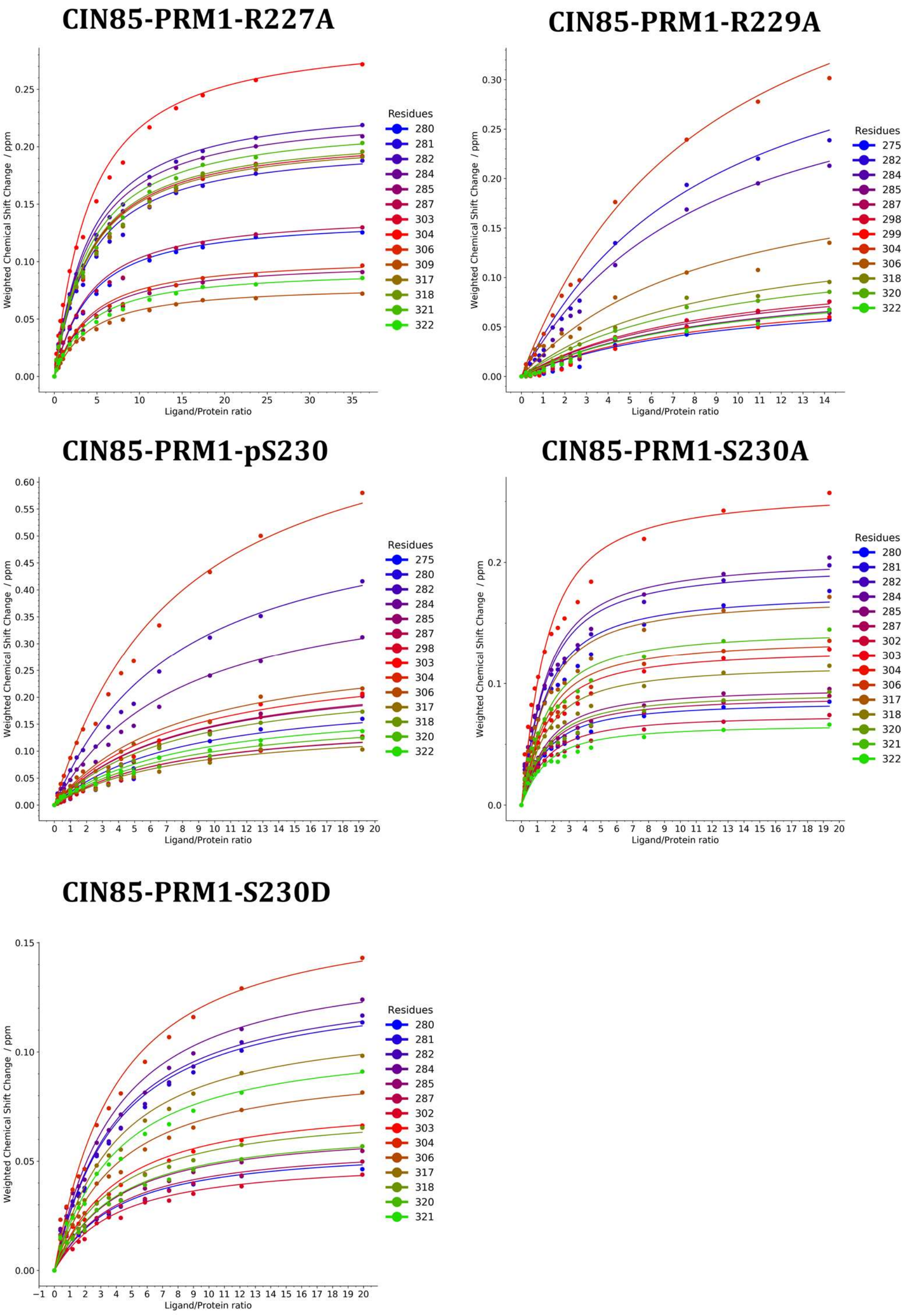

Supplementary Figure S3: Global fits of chemical shift perturbation (CSP) curves obtained after titration of ^{15}N-labeled CIN85 SH3C domain with increasing amounts of CIN85-PRM1 peptides carrying the modifications indicated in the respective insets.

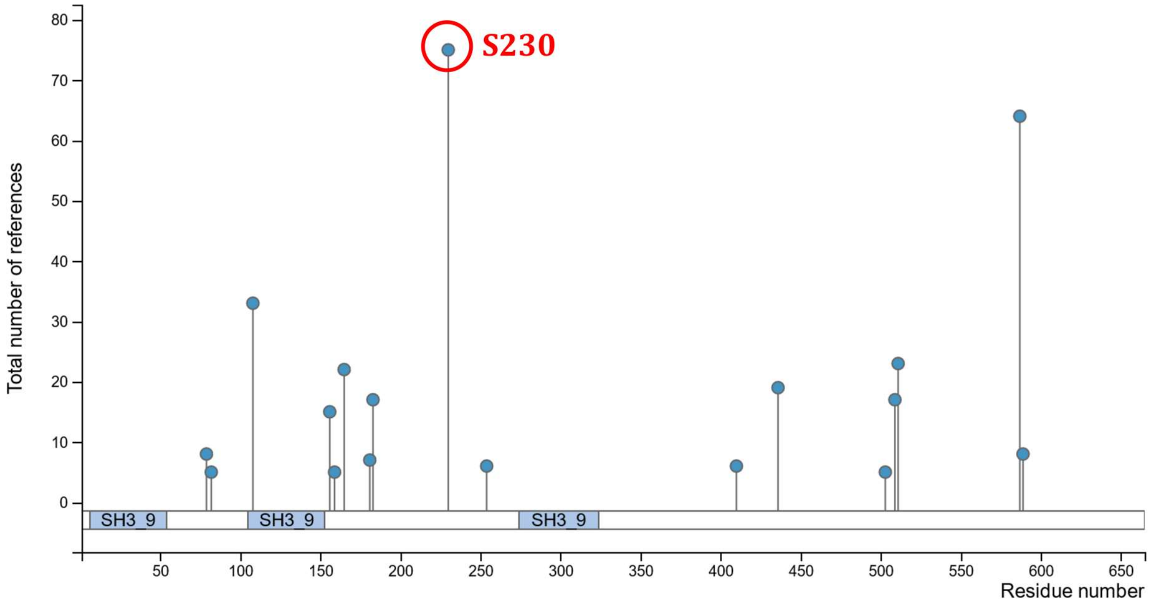

Supplementary Figure S4: Lollipop plot showing known phosphorylation sites of human CIN85 as generated by PhosphoSitePlus® v6.6.0.4[83] for SH3KBP1(human) (Accessed on 2023/08/30 at 17:12 CET). The vertical axis shows the number of publications describing the phosphorylation at the corresponding residue number. Only sites are shown that were mentioned in at least 5 independent studies.

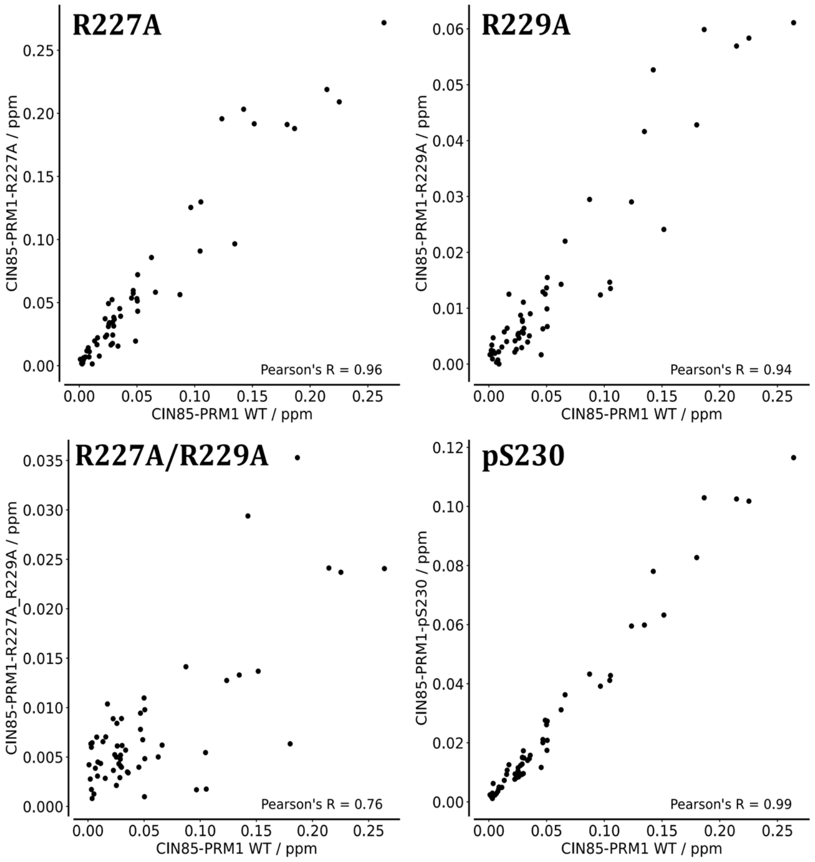

Supplementary Figure S5: Correlation plots of CSP observed for residues inside the SH3C domain for the different peptides compared to the one obtained for the titration of the domain with the wild-type CIN85-PRM1 peptide. The correlation was quantified using Pearson's correlation coefficient.

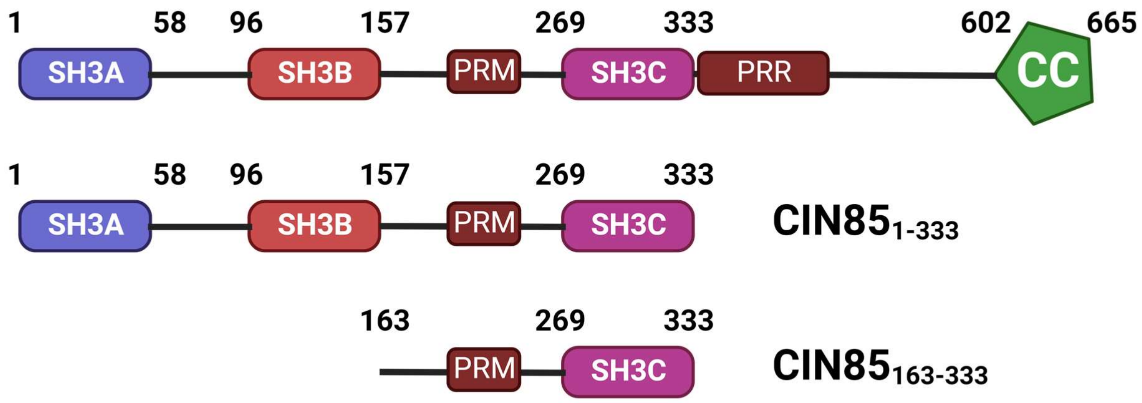

Supplementary Figure S6: Domain boundaries of the CIN85 constructs used throughout the study. The numbers indicate the sequence boundaries of the different functional elements of CIN85. PRM and PRR stand for proline-rich motif and proline-rich region, respectively. CC stands for the C-terminal coiled-coil domain. Created via BioRender.com

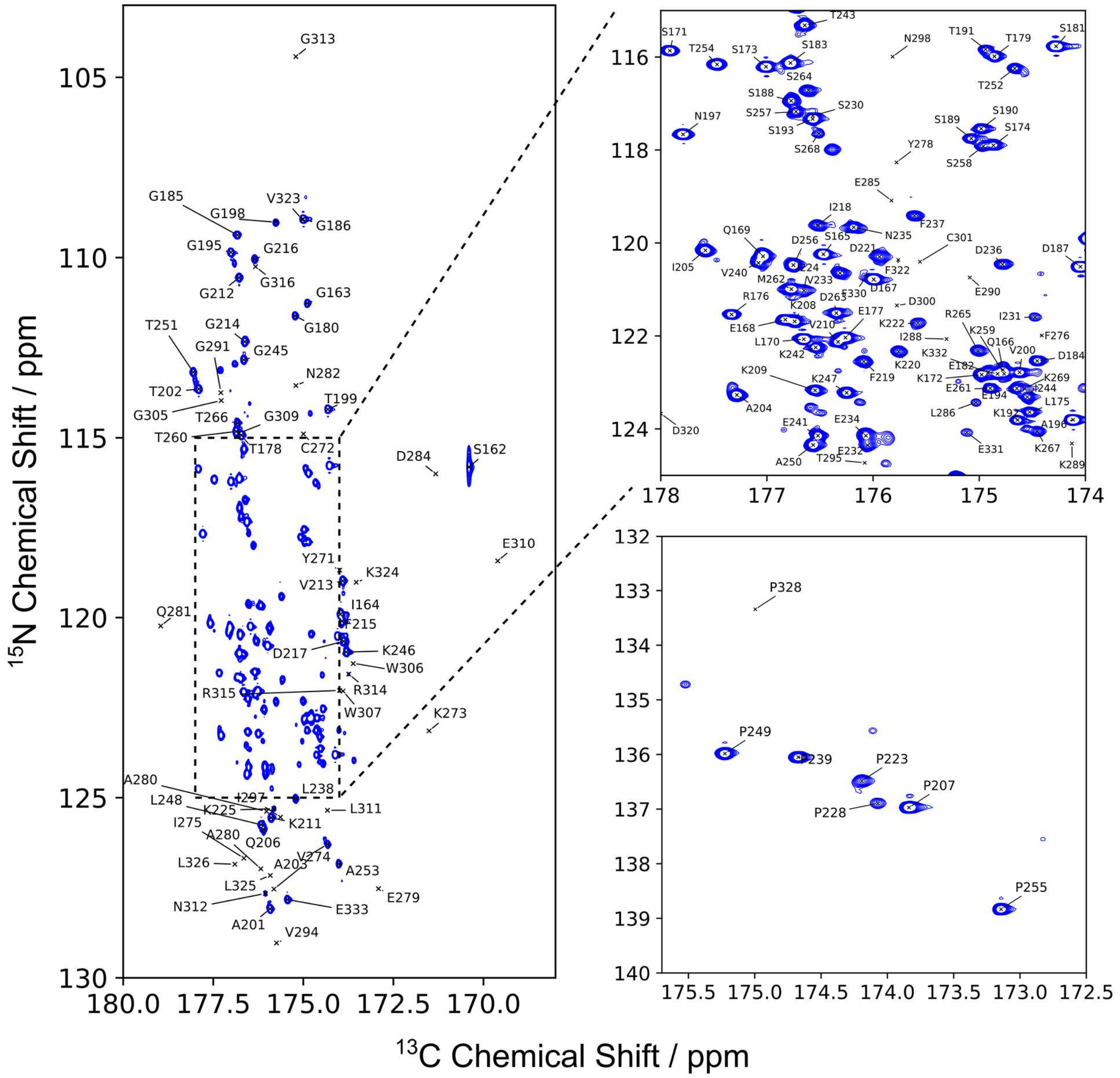

Supplementary Figure S7: Left: ^{13}C-^{15}N-(HACA)CON[24] spectrum of uniformly ^{13}C/^{15}N-labeled CIN85$_{163–333}$ at 1 mM protein concentration (800 MHz, 298 K) with the annotated backbone resonance assignment. **Top right:** Zoom-in on the central crowded region of the (HACA)CON spectrum between 115 and 125 ppm of ^{15}N and 178 and 174 ppm of ^{13}C chemical shifts. **Bottom right:** Zoom-in on the proline region of the (HACA)CON spectrum.

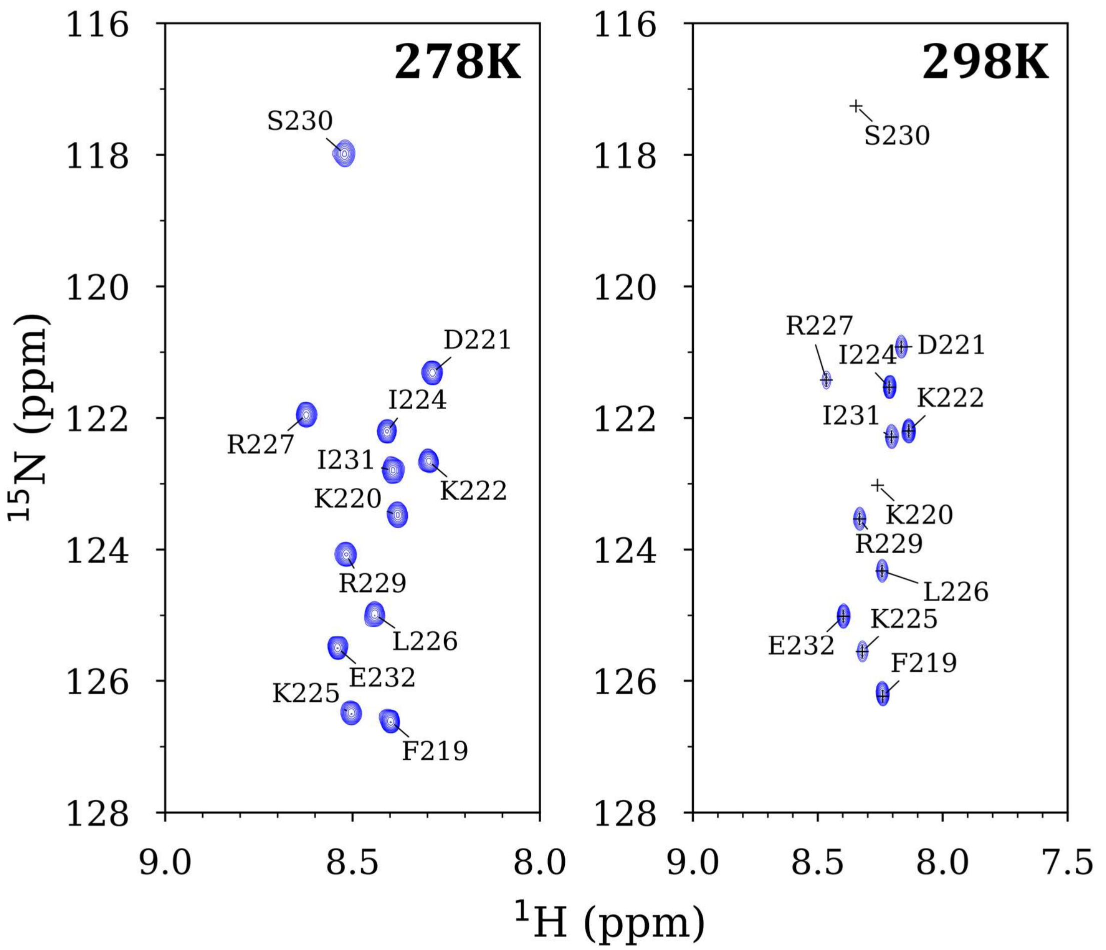

Supplementary Figure S8: Assigned ^{1}H-^{15}N SOFAST-HSQC spectra of CIN85-PRM1 peptide at natural abundance at a temperature of 278 K (left) and 298 K (right). The peptide concentration was 10 mM and the spectra were acquired at 800 MHz.

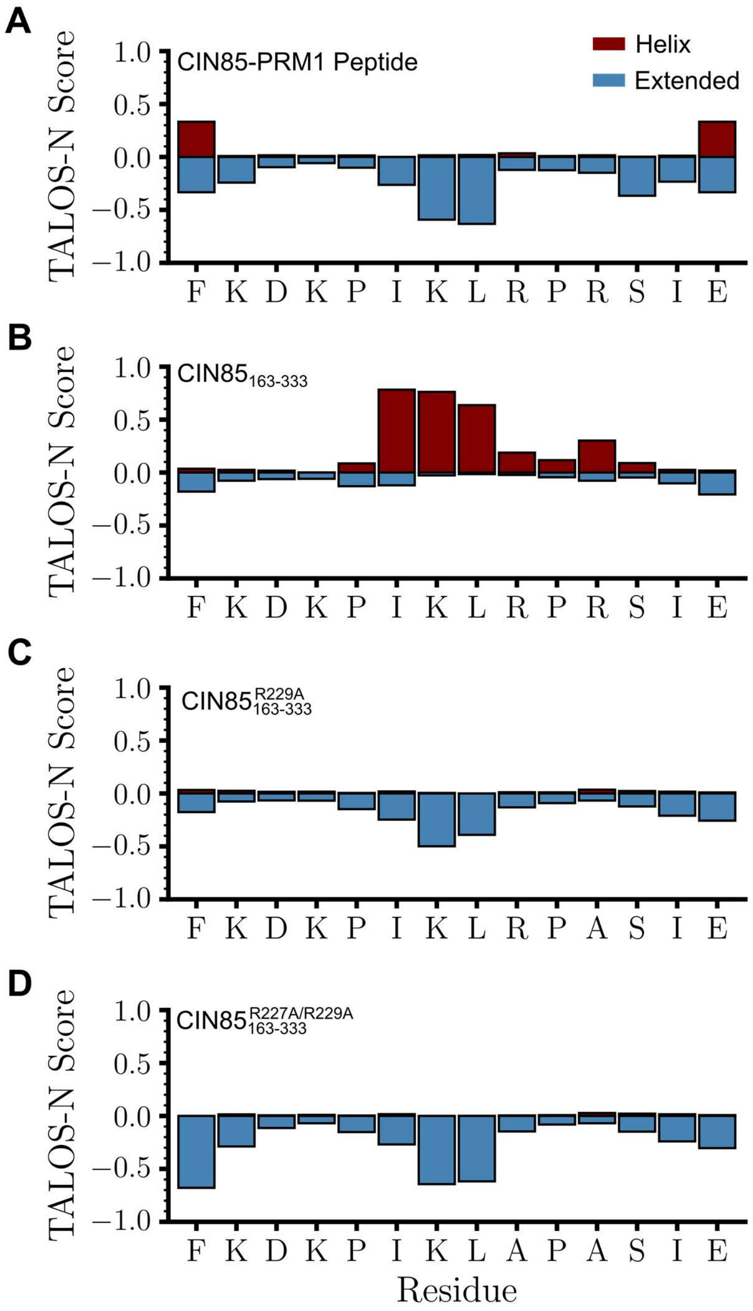

Supplementary Figure S9: Secondary Structure propensities of the residues 219-232 for CIN85-PRM1 (A), $CIN85_{163\text{-}333}$ (B), $CIN85_{163\text{-}333}$-R229A (C) and $CIN85_{163\text{-}333}$-R227A/R229A (D) predicted using TALOS-N[19] based on the assigned chemical shifts.

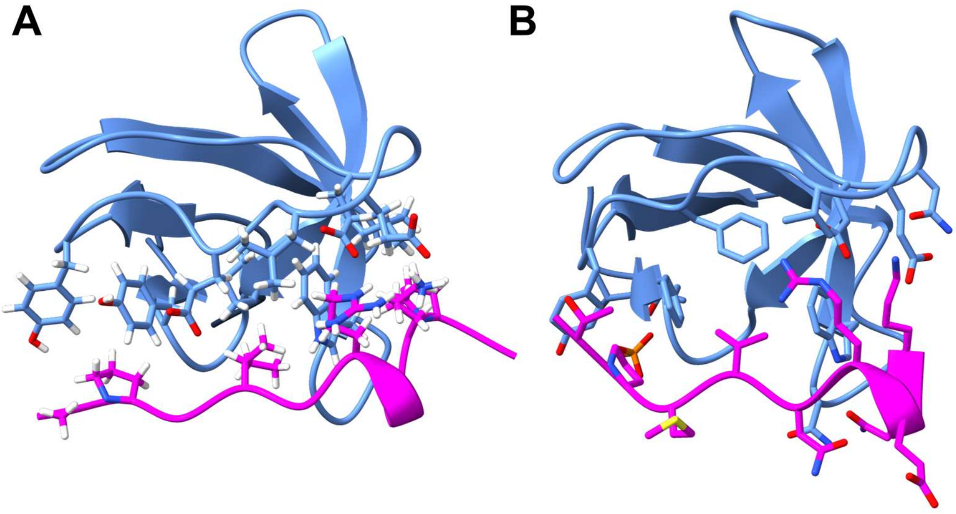

Supplementary Figure S10: Two complexes of SH3 domains and their respective Pro-rich peptides adopting a helical conformation. **A:** Complex between the GADS SH3 domain and a Pro-rich peptide derived from SLP76[25] (PDB: 1h3h). **B:** Complex between the STAM2 SH3 domain and a Pro-rich peptide derived from UBPY[26] (PDB: 1uj0).

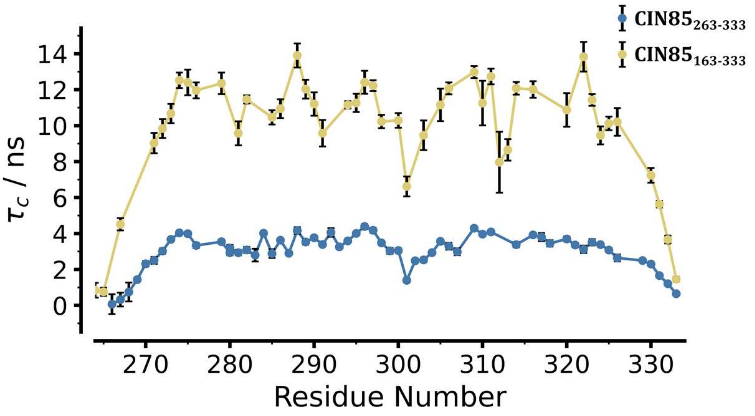

Supplementary Figure S11: Residue-specific apparent τ_c values determined using the TRACT experiment for CIN85$_{163-333}$ (yellow) and CIN85$_{263-333}$ (SH3C, blue). The measurements were acquired at 800 MHz and a temperature of 298 K.

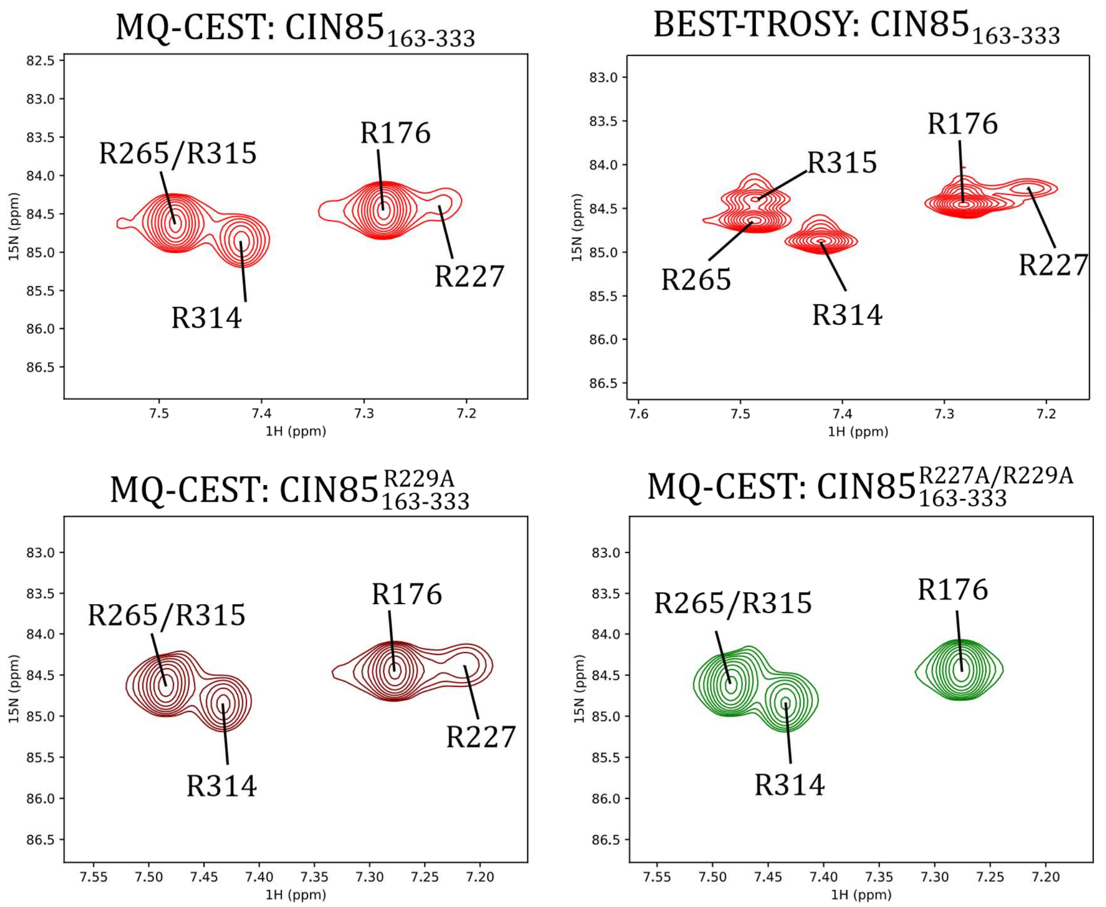

Supplementary Figure S12: Reference planes of the MQ-CEST experiments for the $CIN85_{163\text{-}333}$ constructs. For the WT construct, there is a comparison with the same spectral region of the BEST-TROSY to visualize the overlap of R265 and R315 in the MQ-CEST experiments.

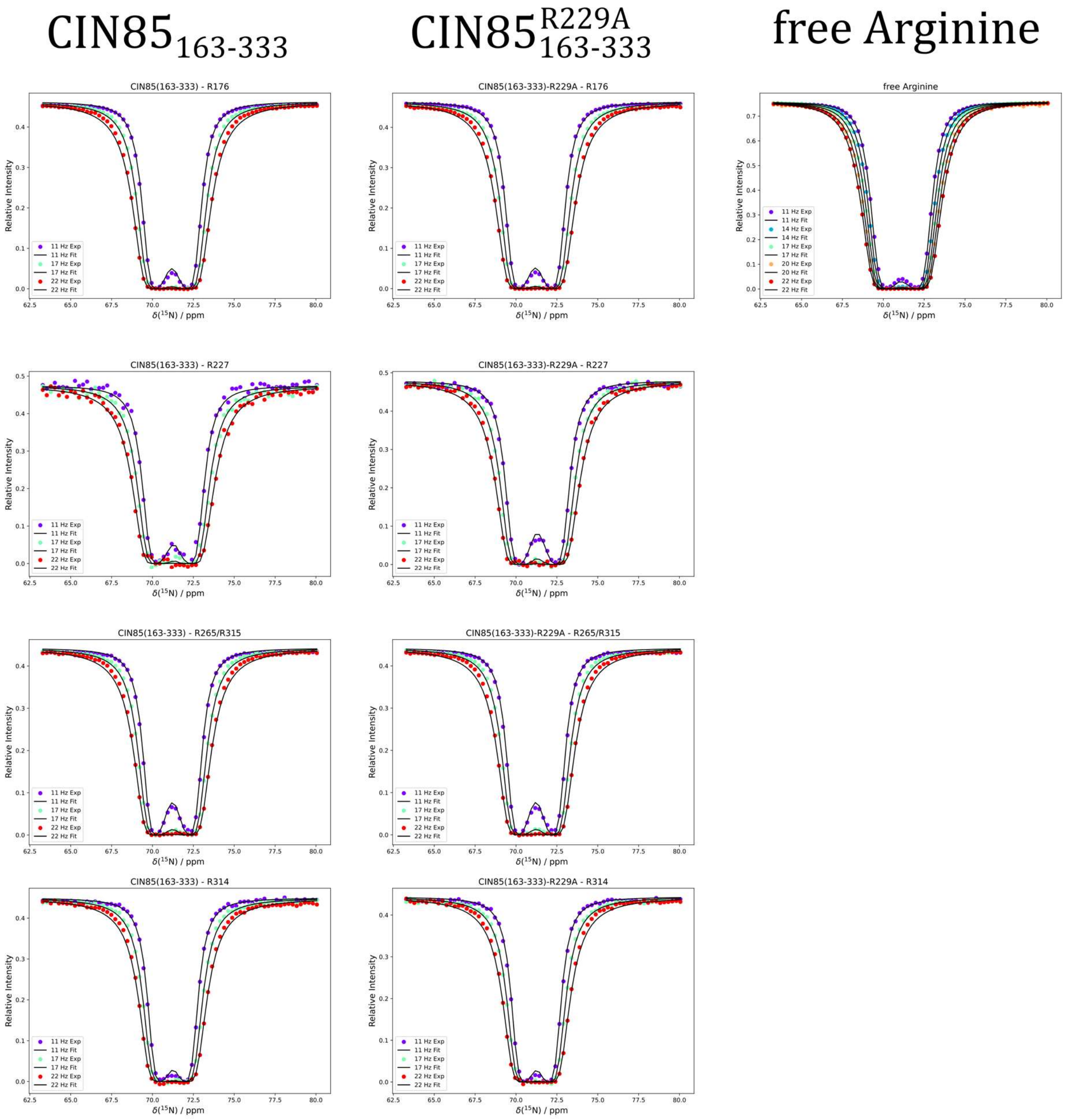

Supplementary Figure S13: MQ-CEST fits of the arginine sidechain peaks visible in the $CIN85_{163\text{-}333}$ constructs (WT and R229A) at 800MHz. For comparison, the MQ-CEST profile of a $^{13}C/^{15}N$-labeled free arginine sample is also shown.

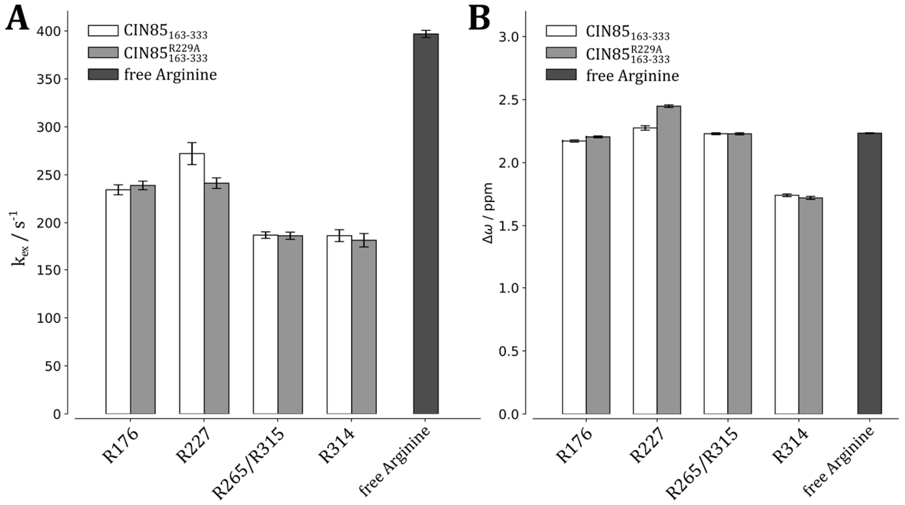

Supplementary Figure S14: Rates of rotation k_{ex} (A) and chemical shift differences $\Delta\omega$ between the two N^{η} nuclei (B) of arginine sidechain guanidinium groups of uniformly $^{13}C/^{15}N$-labeled $CIN85_{163\text{–}333}$, $CIN85_{163\text{–}333}$-R229A and a sample of free arginine as determined via MQ-CEST experiments[16]. Experiments were carried out at 800 MHz and a temperature of 278K.

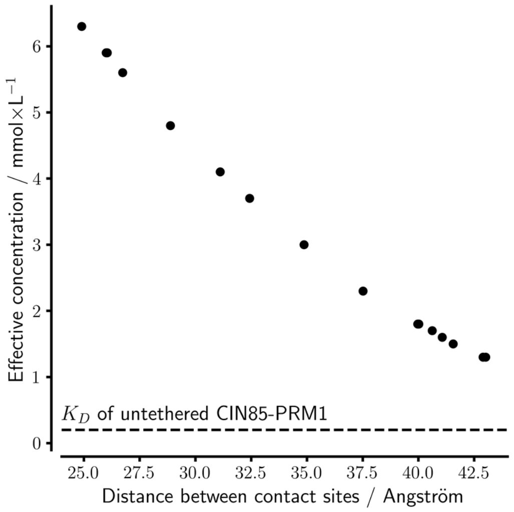

Supplementary Figure S15: Effective concentrations of the SH3C:CIN85-PRM1 complexes. These values were determined via the 'c_{eff} Calculator' web-server in dependence of the distance between contact sites. The distances between residues E232 and K269 used in the calculation were determined from the docked complex structures obtained using HADDOCK. The K_D for the interaction of the untethered CIN85-PRM1 motif to SH3C is indicated as a dashed line (0.2 mM).

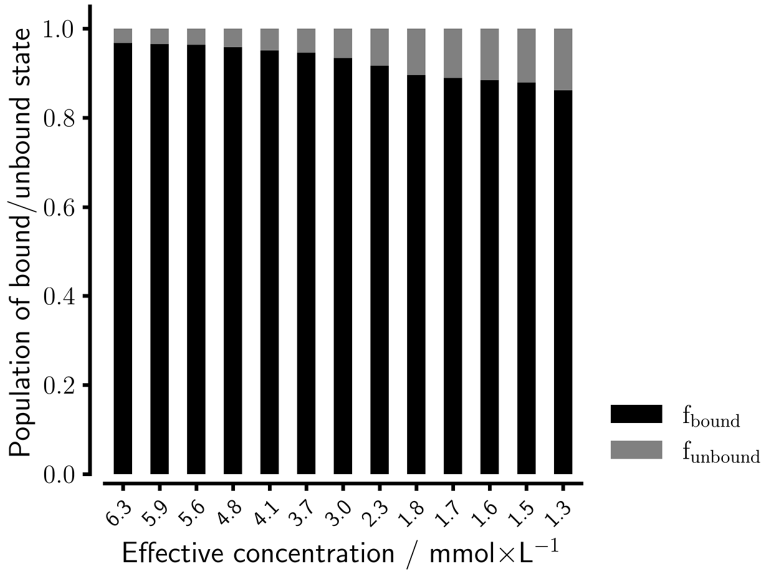

Supplementary Figure S16: Populations of the bound and unbound state of the SH3C:CIN85-PRM1 complex obtained from the effective concentrations. The effective concentrations used in the calculation were taken from Supplementary Figure S15 and the corresponding K_D from the titration of the untethered CIN85-PRM1 peptide to SH3C (see Table 1 in the manuscript).

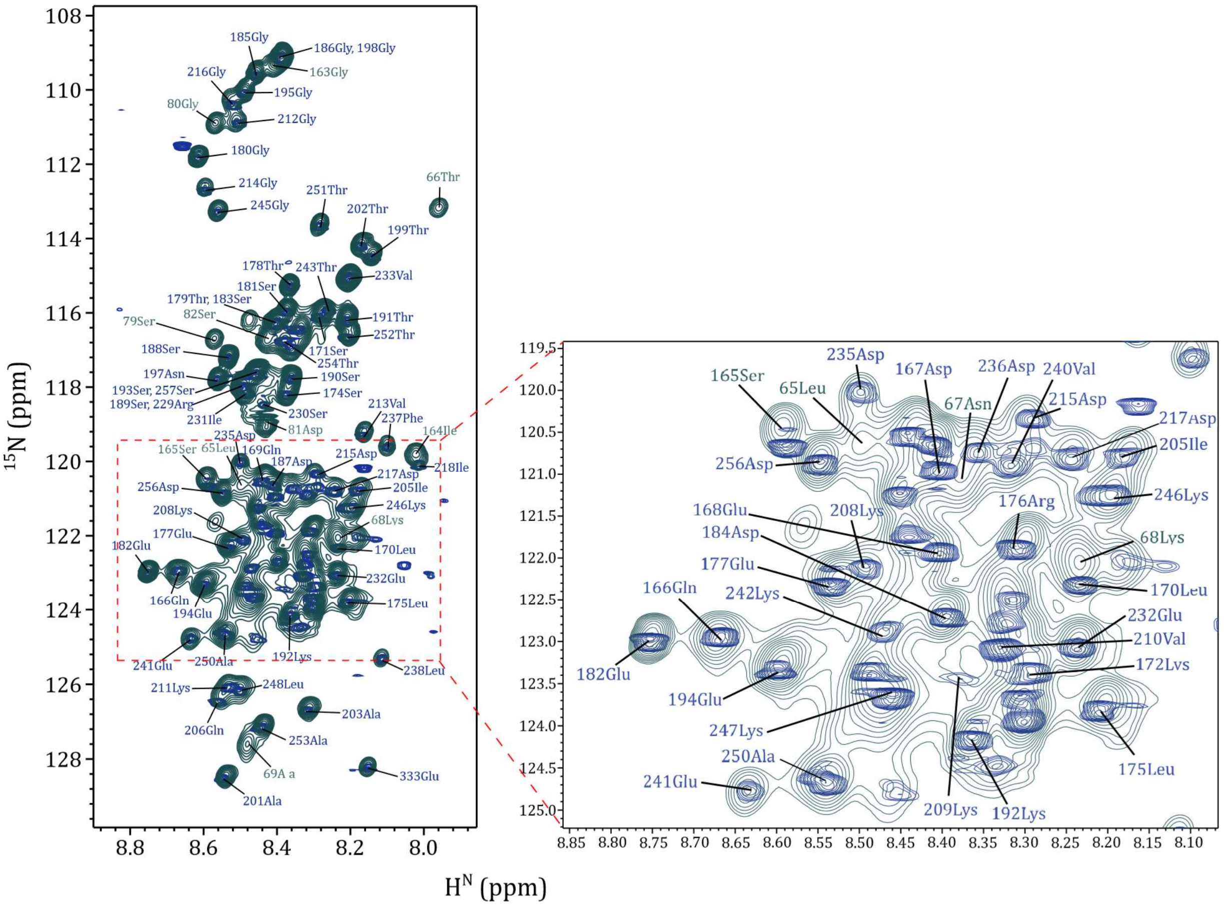

Supplementary Figure S17: Overlay of a ^{1}H–^{15}N TROSY spectrum of 1mM uniformly ^{15}N-^{13}C-labeled CIN85$_{1-333}$ at 1.2 GHz (green) and a ^{1}H–^{15}N SOFAST-HMQC spectrum (blue) of 1 mM unlabeled CIN85$_{163-333}$ at 800 MHz.

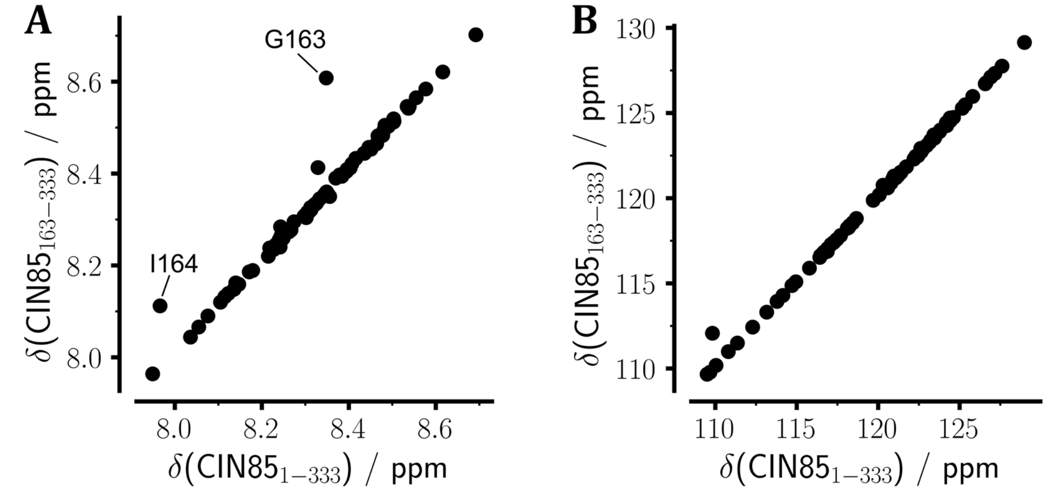

Supplementary Figure S18: Correlation plots of the assigned $^{1}H^{N}$ (A) and $^{15}N^{H}$ (B) chemical shifts of the flexible linker region in $CIN85_{163\text{-}333}$, compared to the assignment obtained using $CIN85_{1\text{-}333}$. Only the $^{1}H^{N}$chemical shifts at the N-terminal end of the construct (G163 and I164) exhibited significantly different chemical shifts from the longer $CIN85_{1\text{-}333}$ construct.

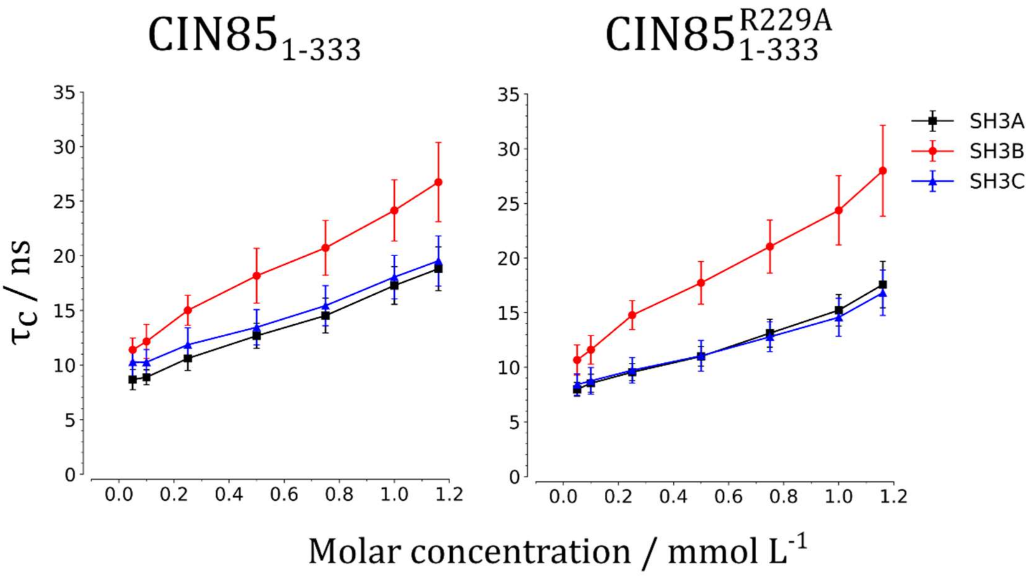

Supplementary Figure S19: Median rotational correlation time of the three SH3 domains of U[^{2}H,^{15}N]-labeled $CIN85_{1\text{–}333}$ (left) and $CIN85_{1\text{-}333}$-R229A (right) calculated from the residue-specific TRACT data, in dependence of the protein concentration. The error bars indicate the spread of the rotational correlation times throughout the SH3 domains, as calculated using the median absolute deviation.

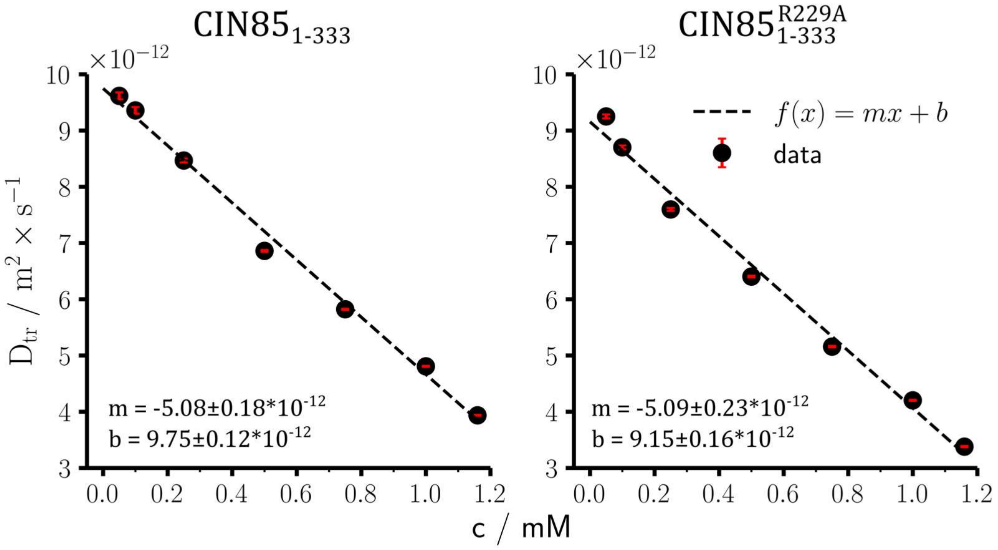

Supplementary Figure S20: Determination of the slope of the concentration-dependence of the diffusion coefficient of $CIN85_{1-333}$ and $CIN85_{1-333}$-R229A via applying a linear fit of the form f(x) = mx + b. Here, m is the slope of the line and b its y-intercept.

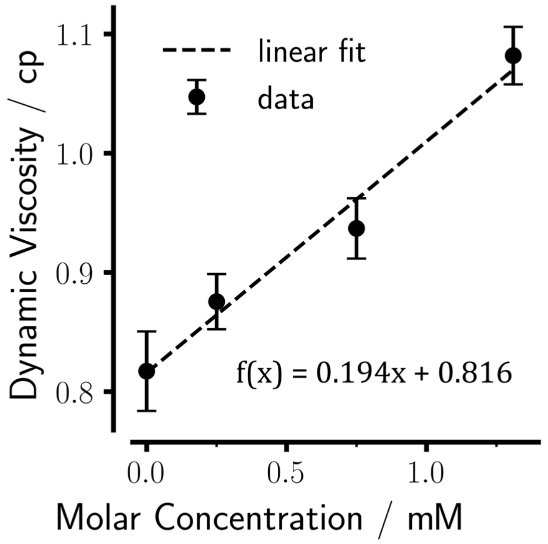

Supplementary Figure S21: Dynamic viscosity of U[^{15}N]-labeled $CIN85_{1-333}$ in the concentration range between 0 and 1.33 mmol L^{-1}. The zero point was just the measurement buffer (20 mM HEPES at pH 7.2, 100 mM NaCl, 1.0 mM TCEP, 0.5 mM Pefabloc, 0.5 mM EDTA, 0.01% NaN_3 and 5% D_2O). Viscosity was monitored via natural abundance ^{17}O T_1 relaxation of water molecules as described elsewhere[27] at 298 K utilizing a Bruker Avance III HD 400MHz spectrometer.

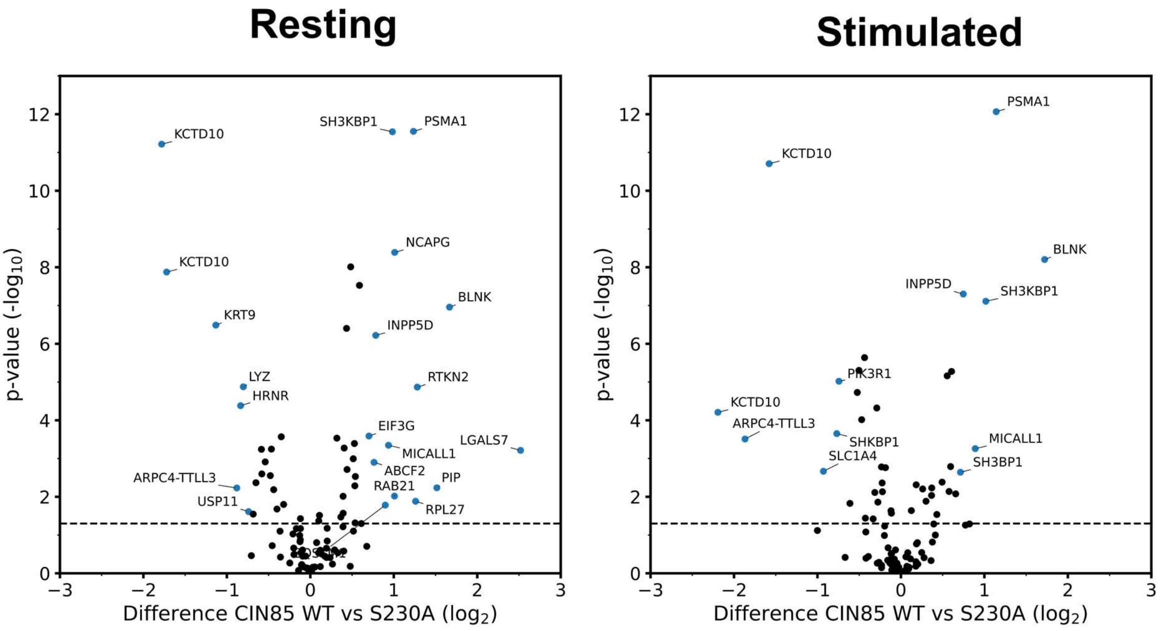

Supplementary Figure S22: Differences in the abundance of peptides purified from DG75 B cells carrying either CIN85 or CIN85-S230A in dependence of the activation state. Protein abundances were quantified using a label-free mass-spectrometric approach. The plots show the difference in the log_2 transformed abundances of proteins between citrine-CIN85 and citrine-CIN85-S230A for the resting (left) and stimulated state (right). Abundances of CIN85-S230A were subtracted from those of CIN85, yielding negative values in case of higher abundance compared to CIN85 and *vice versa*. The y-axis shows the associated p-value and the proteins exhibiting statistically significant differences are labeled by their gene names. The threshold (black dashed line) was set at a p-value of 0.05. In addition to the p-value, a threshold of ± 0.7 was chosen as a criterium for the log_2-transformed difference in abundance.

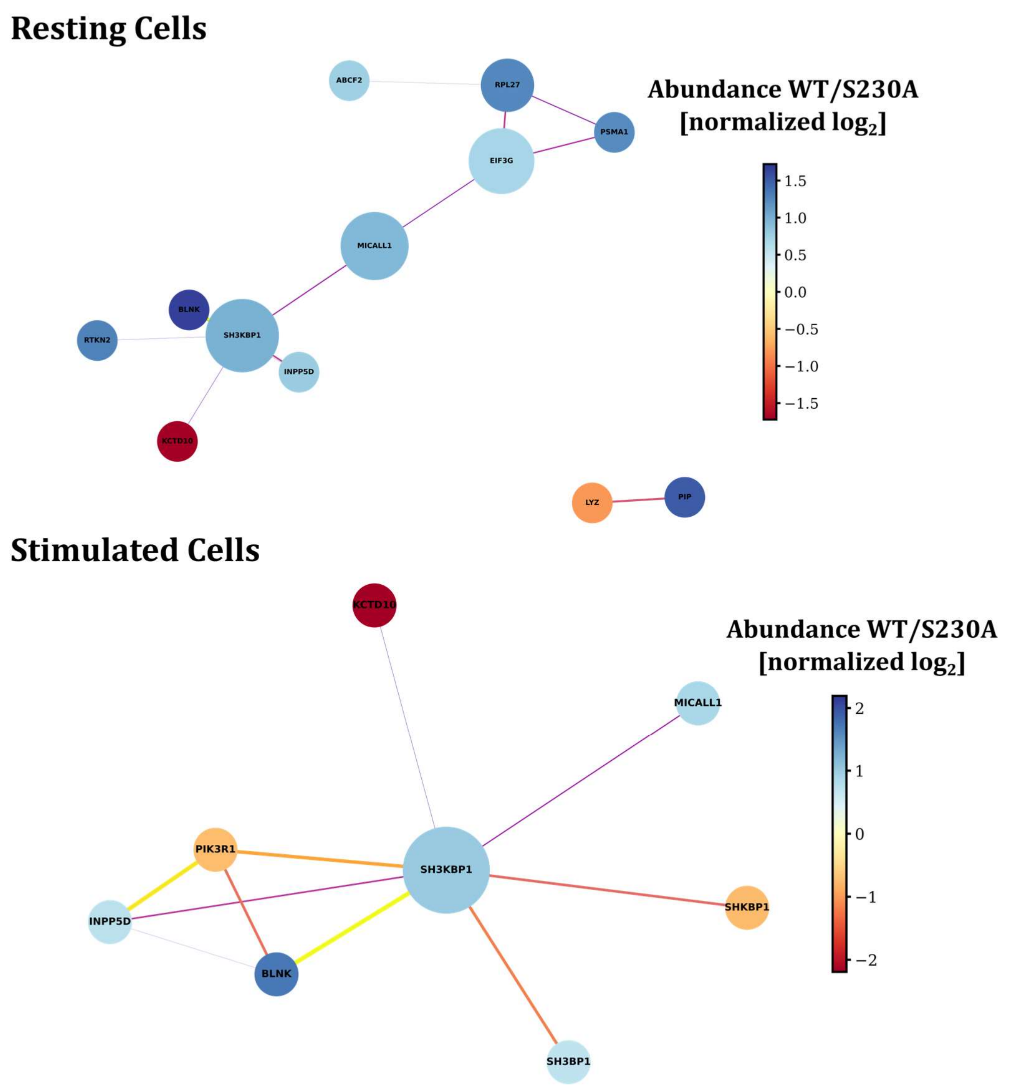

Supplementary Figure S23: STRING-based[28] network analysis based on the proteins that were found to have a significant difference in abundance in the mass-spectrometric experiments (see Supplementary Figure S22). Positive values (white-blue) indicate more abundance in citrine-CIN85 compared to the S230A mutant and negative values (orange-red) reflect a lower abundance in citrine-CIN85. The $\log_2$-transformed values were normalized to show a symmetric range around zero for ease of visualization. The node-diameter reflects the interaction score from STRING. SH3KBP1 is the gene name for CIN85 and BLNK corresponds to SLP65.

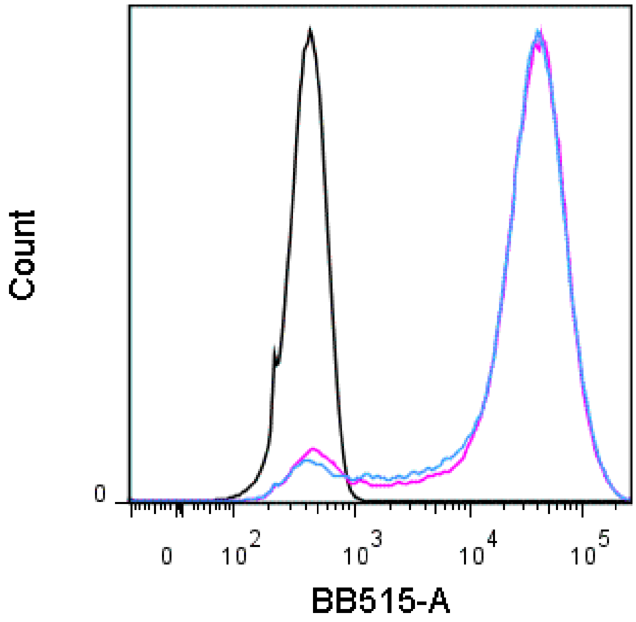

Supplementary Figure S24: Flow-cytometric determination of the expression level of citrine-labeled CIN85 (blue) and citrine-labeled CIN85-S230A (magenta) in DG75 B cells compared to the negative control (black).

Supplementary Tables

Acquisition Parameters of the Resonance Assignment Experiments

Supplementary Table S1: Acquisition Parameters of ^{1}H-detected triple resonance experiments used in the assignment of the CIN85 linker regions. All experiments were part of the standard Bruker library.

Name	TD size(^{1}H/^{15}N/^{13}C)	Scans	%NUS	Field / MHz
CIN85$_{1\text{-}333}$				
BEST-TROSY-HNCA	2332/204/256	88	5	1200
BEST-TROSY-HN(CO)CA	2332/212/256	88	4	1200
BEST-TROSY-HNCO	1750/204/156	32	4	800
CIN85$_{163\text{-}333}$				
BEST-TROSY-HNCACB	1958/96/200	128	10	700
BEST-TROSY-HN(CO)CACB	1958/96/200	128	7.8	700

Supplementary Table S2: Acquisition Parameters of ^{13}C-detected triple resonance experiments used in the assignment of the CIN85 linker regions.

Name	TD size (^{13}C/^{15}N/X)	Scans	%NUS	Field / MHz
CIN85$_{163\text{-}333}$				
(HACA)N(CA)CON[29]	2048/80/108(^{15}N)	64	8.2	700
(H)CCCON[30]	2048/80/128(^{13}C)	16	10	700
H(CC)CON[31]	2048/44/120(^{1}H)	16	8	900
C-HNCO[32]	1024/90/180(^{1}H)	64	10	800
CIN85$_{163\text{-}333}$-R229A				
(HACA)N(CA)CON[29]	2048/80/128(^{15}N	80	8.1	700
(H)CCCON[30]	2048/80/128(^{13}C)	16	8.5	700
H(CC)CON[31]	2048/44/120(^{1}H)	16	8.5	700
C-HNCO[32]	2048/84/112(^{1}H)	56	9.7	700
CIN85$_{163\text{-}333}$-R227A/R229A				
(HACA)N(CA)CON[29]	2048/80/118(^{15}N	48	8.1	700
(H)CCCON[30]	2070/80/128(^{13}C)	16	8.5	800

H(CC)CON[31]	2376/80/128(^{1}H)	16	8.5	800
C-HNCO[32]	2048/84/112(^{1}H)	32	9.7	700

Experiment Definitions of ^{13}C-Detected Resonance Assignment Experiments for the Semi-Automatic Assignment using FLYA

SPECTRUM hacaCON CO N

0.980 CO:C_BYL (C_ALI N_AMI) N:N_AMI

SPECTRUM hacaNcaCON N_1 N_2 CO

0.980 CO:C_BYL N_1:N_AMI C_ALI C_BYL

0.980 N_1:N_AMI C_ALI CO:C_BYL N_2:N_AMI

SPECTRUM hCCCON CO N C

0.98 N:N_AMI (C_ALI) CO:C_BYL C:C_ALI

0.98 N:N_AMI (C_ALI) CO:C_BYL C_ALI C:C_ALI

0.80 N:N_AMI (C_ALI) CO:C_BYL C_ALI C_ALI C:C_ALI

0.60 N:N_AMI (C_ALI) CO:C_BYL C_ALI C_ALI C_ALI C:C_ALI

0.40 N:N_AMI (C_ALI) CO:C_BYL C_ALI C_ALI C_ALI C_ALI C:C_ALI

SPECTRUM HccCON CO N H

0.98 N:N_AMI (C_ALI) CO:C_BYL C_ALI H:H_ALI

0.98 N:N_AMI (C_ALI) CO:C_BYL C_ALI C_ALI H:H_ALI

0.80 N:N_AMI (C_ALI) CO:C_BYL C_ALI C_ALI C_ALI H:H_ALI

0.60 N:N_AMI (C_ALI) CO:C_BYL C_ALI C_ALI C_ALI C_ALI H:H_ALI

0.40 N:N_AMI (C_ALI) CO:C_BYL C_ALI C_ALI C_ALI C_ALI C_ALI H:H_ALI

SPECTRUM CBCACON CO N C

0.98 N:N_AMI (C_ALI) CO:C_BYL C:C_ALI

0.98 N:N_AMI (C_ALI) CO:C_BYL C_ALI C:C_ALI

SPECTRUM CONH CO N H

0.98 CO:C_BYL (C_ALI N_AMI) N:N_AMI H:H_AMI

References

1. Wong, L. E.; Bhatt, A.; Erdmann, P. S.; Hou, Z.; Maier, J.; Pirkuliyeva, S.; Engelke, M.; Becker, S.; Plitzko, J.; Wienands, J.; Griesinger, C., Tripartite phase separation of two signal effectors with vesicles priming B cell responsiveness. *Nat. Commun.* **2020,** *11* (1), 848.
2. Oellerich, T.; Grønborg, M.; Neumann, K.; Hsiao, H.-H.; Urlaub, H.; Wienands, J., SLP-65 Phosphorylation Dynamics Reveals a Functional Basis for Signal Integration by Receptor-proximal Adaptor Proteins*. *Mol. Cell Proteomics* **2009,** *8* (7), 1738-1750.
3. Oellerich, T.; Bremes, V.; Neumann, K.; Bohnenberger, H.; Dittmann, K.; Hsiao, H.-H.; Engelke, M.; Schnyder, T.; Batista, F. D.; Urlaub, H.; Wienands, J., The B-cell antigen receptor signals through a preformed transducer module of SLP65 and CIN85. *EMBO J* **2011,** *30* (17), 3620-3634.
4. Atanassov, I.; Urlaub, H., Increased proteome coverage by combining PAGE and peptide isoelectric focusing: Comparative study of gel-based separation approaches. *PROTEOMICS* **2013,** *13* (20), 2947-2955.
5. Delaglio, F.; Grzesiek, S.; Vuister, G.; Zhu, G.; Pfeifer, J.; Bax, A., NMRPipe: A multidimensional spectral processing system based on UNIX pipes. *J. Biomol. NMR* **1995,** *6* (3).
6. Auguin, D.; Barthe, P.; Royer, C.; Stern, M.-H.; Noguchi, M.; Arold, S. T.; Roumestand, C., Structural Basis for the Co-activation of Protein Kinase B by T-cell Leukemia-1 (TCL1) Family Proto-oncoproteins. *J. Biol. Chem.* **2004,** *279* (34), 35890-35902.
7. Newville, M.; Stensitzki, T.; Allen, D. B.; Ingargiola, A., LMFIT: Non-Linear Least-Square Minimization and Curve-Fitting for Python. 2014.
8. Ying, J.; Delaglio, F.; Torchia, D. A.; Bax, A., Sparse multidimensional iterative lineshape-enhanced (SMILE) reconstruction of both non-uniformly sampled and conventional NMR data. *J. Biomol. NMR* **2016,** *68* (2), 101-118.
9. Hyberts, S. G.; Milbradt, A. G.; Wagner, A. B.; Arthanari, H.; Wagner, G., Application of iterative soft thresholding for fast reconstruction of NMR data non-uniformly sampled with multidimensional Poisson Gap scheduling. *J. Biomol. NMR* **2012,** *52* (4), 315-327.
10. Lee, W.; Rahimi, M.; Lee, Y.; Chiu, A., POKY: a software suite for multidimensional NMR and 3D structure calculation of biomolecules. *Bioinformatics* **2021,** *37* (18), 3041-3042.
11. Schmidt, E.; Güntert, P., A New Algorithm for Reliable and General NMR Resonance Assignment. *J. Am. Chem. Soc.* **2012,** *134* (30), 12817-12829.

12. Kühn, J.; Wong, L. E.; Pirkuliyeva, S.; Schulz, K.; Schwiegk, C.; Fünfgeld, K. G.; Keppler, S.; Batista, F. D.; Urlaub, H.; Habeck, M.; Becker, S.; Griesinger, C.; Wienands, J., The adaptor protein CIN85 assembles intracellular signaling clusters for B cell activation. *Sci. Signal.* **2016,** *9* (434), 66.
13. Lee, D.; Hilty, C.; Wider, G.; Wuthrich, K., Effective rotational correlation times of proteins from NMR relaxation interference. *J. Magn. Reson.* **2006,** *178* (1), 72-6.
14. Robson, S. A.; Dag, C.; Wu, H.; Ziarek, J. J., TRACT revisited: an algebraic solution for determining overall rotational correlation times from cross-correlated relaxation rates. *J. Biomol. NMR* **2021,** *75* (8), 293-302.
15. Horst, R.; Horwich, A. L.; Wüthrich, K., Translational Diffusion of Macromolecular Assemblies Measured Using Transverse-Relaxation-Optimized Pulsed Field Gradient NMR. *J. Am. Chem. Soc.* **2011,** *133* (41), 16354-16357.
16. Karunanithy, G.; Reinstein, J.; Hansen, D. F., Multiquantum Chemical Exchange Saturation Transfer NMR to Quantify Symmetrical Exchange: Application to Rotational Dynamics of the Guanidinium Group in Arginine Side Chains. *J. Phys. Chem. Lett.* **2020,** *11* (14), 5649-5654.
17. Guenneugues, M.; Berthault, P.; Desvaux, H., A Method for DeterminingB1Field Inhomogeneity. Are the Biases Assumed in Heteronuclear Relaxation Experiments Usually Underestimated? *J. Magn. Reson.* **1999,** *136* (1), 118-126.
18. Kjaergaard, M.; Glavina, J.; Chemes, L. B., Predicting the effect of disordered linkers on effective concentrations and avidity with the ceff calculator app. In *Linkers in Biomacromolecules*, 2021; pp 145-171.
19. Shen, Y.; Bax, A., Protein backbone and sidechain torsion angles predicted from NMR chemical shifts using artificial neural networks. *J. Biomol. NMR* **2013,** *56* (3), 227-241.
20. Honorato, R. V.; Koukos, P. I.; Jimnez-Garcia, B.; Tsaregorodtsev, A.; Verlato, M.; Giachetti, A.; Rosato, A.; Bonvin, A. M. J. J., Structural Biology in the Clouds: The WeNMR-EOSC Ecosystem. *Front. Mol. Biosci.* **2021,** *8*.
21. van Zundert, G. C. P.; Rodrigues, J. P. G. L. M.; Trellet, M.; Schmitz, C.; Kastritis, P. L.; Karaca, E.; Melquiond, A. S. J.; van Dijk, M.; de Vries, S. J.; Bonvin, A. M. J. J., The HADDOCK2.2 Web Server: User-Friendly Integrative Modeling of Biomolecular Complexes. *J. Mol. Biol.* **2016,** *428* (4), 720-725.
22. Sievers, F.; Wilm, A.; Dineen, D.; Gibson, T. J.; Karplus, K.; Li, W.; Lopez, R.; McWilliam, H.; Remmert, M.; Söding, J.; Thompson, J. D.; Higgins, D. G., Fast, scalable generation of high-quality protein multiple sequence alignments using Clustal Omega. *Mol. Syst. Biol.* **2011,** *7* (1), 539.
23. Altschul, S. F.; Gish, W.; Miller, W.; Myers, E. W.; Lipman, D. J., Basic local alignment search tool. *J. Mol. Biol.* **1990,** *215* (3), 403-410.
24. Bastidas, M.; Gibbs, E. B.; Sahu, D.; Showalter, S. A., A primer for carbon-detected NMR applications to intrinsically disordered proteins in solution. *Concepts Magn Reson Part A* **2015,** *44* (1), 54-66.
25. Liu, Q.; Berry, D.; Nash, P.; Pawson, T.; McGlade, C. J.; Li, S. S., Structural basis for specific binding of the Gads SH3 domain to an RxxK motif-containing SLP-76 peptide: a novel mode of peptide recognition. *Molecular cell* **2003,** *11* (2), 471-81.

26. Kaneko, T.; Kumasaka, T.; Ganbe, T.; Sato, T.; Miyazawa, K.; Kitamura, N.; Tanaka, N., Structural Insight into Modest Binding of a Non-PXXP Ligand to the Signal Transducing Adaptor Molecule-2 Src Homology 3 Domain. *J. Biol. Chem.* **2003,** *278* (48), 48162-48168.
27. Rezaei-Ghaleh, N.; Munari, F.; Becker, S.; Assfalg, M.; Griesinger, C., A facile oxygen-17 NMR method to determine effective viscosity in dilute, molecularly crowded and confined aqueous media. *Chem. Commun.* **2019,** *55* (82), 12404-12407.
28. Szklarczyk, D.; Gable, A. L.; Nastou, K. C.; Lyon, D.; Kirsch, R.; Pyysalo, S.; Doncheva, N. T.; Legeay, M.; Fang, T.; Bork, P.; Jensen, L. J.; von Mering , C., The STRING database in 2021: customizable protein–protein networks, and functional characterization of user-uploaded gene/measurement sets. *Nucleic Acids Res.* **2020,** *49* (D1), D605-D612.
29. Cook, E. C.; Usher, G. A.; Showalter, S. A., Chapter Five - The Use of 13C Direct-Detect NMR to Characterize Flexible and Disordered Proteins. In *Meth. Enzymol.*, Rhoades, E., Ed. Academic Press: 2018; Vol. 611, pp 81-100.
30. Bermel, W.; Bertini, I.; Felli, I. C.; Kümmerle, R.; Pierattelli, R., Novel 13C direct detection experiments, including extension to the third dimension, to perform the complete assignment of proteins. *J. Magn. Reson.* **2006,** *178* (1), 56-64.
31. O'Hare, B.; Benesi, A. J.; Showalter, S. A., Incorporating 1H chemical shift determination into 13C-direct detected spectroscopy of intrinsically disordered proteins in solution. *J. Magn. Reson.* **2009,** *200* (2), 354-358.
32. Bermel, W.; Bertini, I.; Duma, L.; Felli, I. C.; Emsley, L.; Pierattelli, R.; Vasos, P. R., Complete Assignment of Heteronuclear Protein Resonances by Protonless NMR Spectroscopy. *Angew. Chem. Int. Ed.* **2005,** *44* (20), 3089-3092.
